# Endoscopic diagnosis and treatment of esophageal adenocarcinoma: introduction of Japan Esophageal Society classification of Barrett’s esophagus

**DOI:** 10.1007/s00535-018-1491-x

**Published:** 2018-06-30

**Authors:** Ryu Ishihara, Kenichi Goda, Tsuneo Oyama

**Affiliations:** 1 Department of Gastrointestinal Oncology, Osaka International Cancer Institute, 1-69 Otemae 3-chome, Chuo-ku, Osaka, 541-8567 Japan; 2 Digestive Disease Centre, Showa University, Koto-Toyosu Hospital, Tokyo, Japan; 3 Department of Endoscopy, Saku Central Hospital Advanced Care Center, Saku, Japan

**Keywords:** Endoscopic diagnosis, Endoscopic treatment, Esophageal adenocarcinoma, Barrett’s esophagus

## Abstract

Endoscopic surveillance of Barrett’s esophagus has become a foundation of the management of esophageal adenocarcinoma (EAC). Surveillance for Barrett’s esophagus commonly involves periodic upper endoscopy with biopsies of suspicious areas and random four-quadrant biopsies. However, targeted biopsies using narrow-band imaging can detect more dysplastic areas and thus reduce the number of biopsies required. Several specific mucosal and vascular patterns characteristic of Barrett’s esophagus have been described, but the proposed criteria are complex and diverse. Simpler classifications have recently been developed focusing on the differentiation between dysplasia and non-dysplasia. These include the Japan Esophageal Society classification, which defines regular and irregular patterns in terms of mucosal and vascular shapes. Cancer invasion depth is diagnosed by endoscopic ultrasonography (EUS); however, a meta-analysis of EUS staging of superficial EAC showed favorable pooled values for mucosal cancer staging, but unsatisfactory diagnostic results for EAC at the esophagogastric junction. Endoscopic resection has recently been suggested as a more accurate staging modality for superficial gastrointestinal cancers than EUS. Following endoscopic resection for gastrointestinal cancers, the risk of metastasis can be evaluated based on the histology of the resected specimen. European guidelines describe endoscopic resection as curative for well- or moderately differentiated mucosal cancers without lymphovascular invasion, and these criteria might be extended to lesions invading the submucosa (≤ 500 μm), i.e., to low-risk, well- or moderately differentiated tumors without lymphovascular involvement, and < 3 cm. These criteria were confirmed by a recent study in Japan.

## Introduction

Esophageal adenocarcinoma (EAC) is an aggressive disease with an increasing incidence in the Western world [1–3]. Although no equivalent data are available for Eastern countries, the rate of EAC is expected to increase in Asia because of the decreasing prevalence of *Helicobacter pylori* infection and Westernization of the diet [4, 5]. Survival of patients with EAC correlates with disease stage, with a 5-year-survival rate of about 20% in patients with locally advanced disease [6]. The poor survival of patients with advanced EAC indicates the need for its early detection [7, 8]. Endoscopic surveillance of Barrett’s esophagus (BE) has become a foundation of the management of EAC, especially in Western countries [9–11], and this trend has accelerated in line with recent developments in advanced imaging and endoscopic resection technologies.

## Surveillance and classification of lesions in patients with BE

Surveillance for EAC in patients with BE commonly involves periodic upper endoscopy, with biopsies of suspicious areas and random four-quadrant biopsies [12]. However, this biopsy protocol is time consuming, carries a risk of sampling error, and is hampered by low patient compliance [13]. New endoscopic techniques have, therefore, been developed to improve the recognition of specialized intestinal metaplasia (SIM), dysplasia, and cancer, by enhancing mucosal morphology. The most widely used such modality is narrow-band imaging (NBI) [14], and targeted biopsies sampled by this method allowed the detection of more dysplastic areas, therefore, reducing the number of biopsies required [15].

Several groups have described specific mucosal and vascular patterns characteristic for the diagnosis of lesions in BE using NBI [16–22]. These classification systems suggested that irregular mucosal pattern and vessels are predictive of dysplasia, while a ridged/villous pattern is predictive of SIM; however, despite promising initial findings, subsequent validation studies of these classification systems have reported unfavorable results [23–27]. Furthermore, the proposed criteria were complex and diverse, thus limiting their use in daily clinical practice, with the complexity associated with the concept of differentiating between SIM and non-SIM and between dysplasia and non-dysplasia within the same classification.

Simpler classifications have recently been developed focusing on differentiating between dysplasia and non-dysplasia, with the aim of improving the clinical utility of the classification [28, 29]. The new classifications classify most mucosal or vascular descriptors as “regular” for non-dysplastic and “irregular” for dysplastic BE (Table 1). These simple descriptors make the classifications easy to apply in clinical practice, with acceptable sensitivity, specificity, and inter-observer agreement for the diagnosis of dysplasia in BE (Table 1).

**Table 1:** New endoscopic classifications for the diagnosis of lesions in patients with Barrett’s esophagus

	BING classification	JES classification for Barrett’s esophagus
Non-dysplasia	Mucosal pattern: regular	Mucosal pattern: regular
	Vascular pattern: regular	Vascular pattern: regular flat pattern
Dysplasia	Mucosal pattern: absent or irregular	Mucosal pattern: irregular
	Vascular pattern: irregular	Vascular pattern: irregular
Diagnostic accuracy	Sensitivity 80%	Sensitivity 87%
	Specificity 88%	Specificity 97%
Reproducibility	*κ* = 0.68	*κ* = 0.77

*BING* Barrett’s International NBI Group, *JES* Japan Esophageal Society

**Fig. 1:**
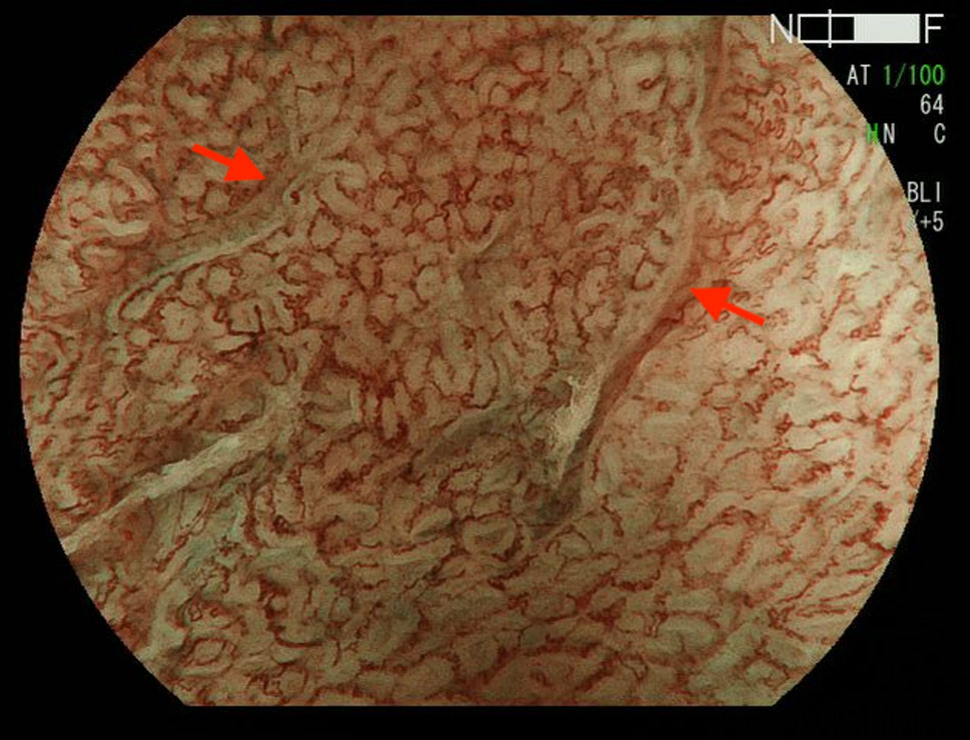
Barrett’s esophageal cancer showing irregular vascular pattern (net type)

**Fig. 2:**
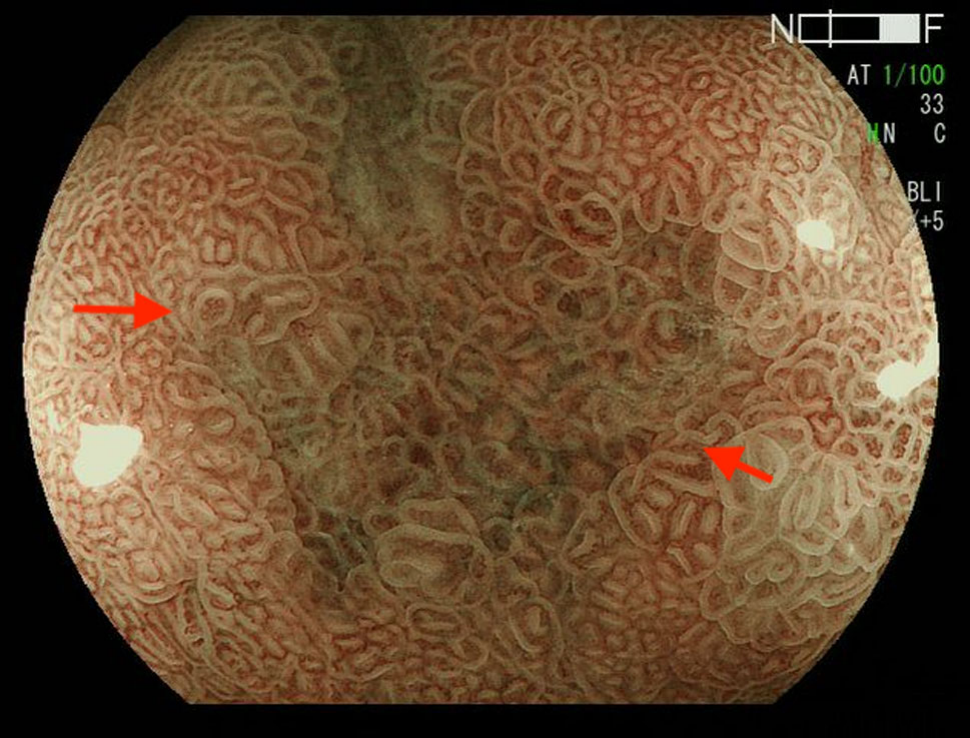
Barrett’s esophageal cancer showing irregular mucosal pattern (non-pit type)

**Fig. 3:**
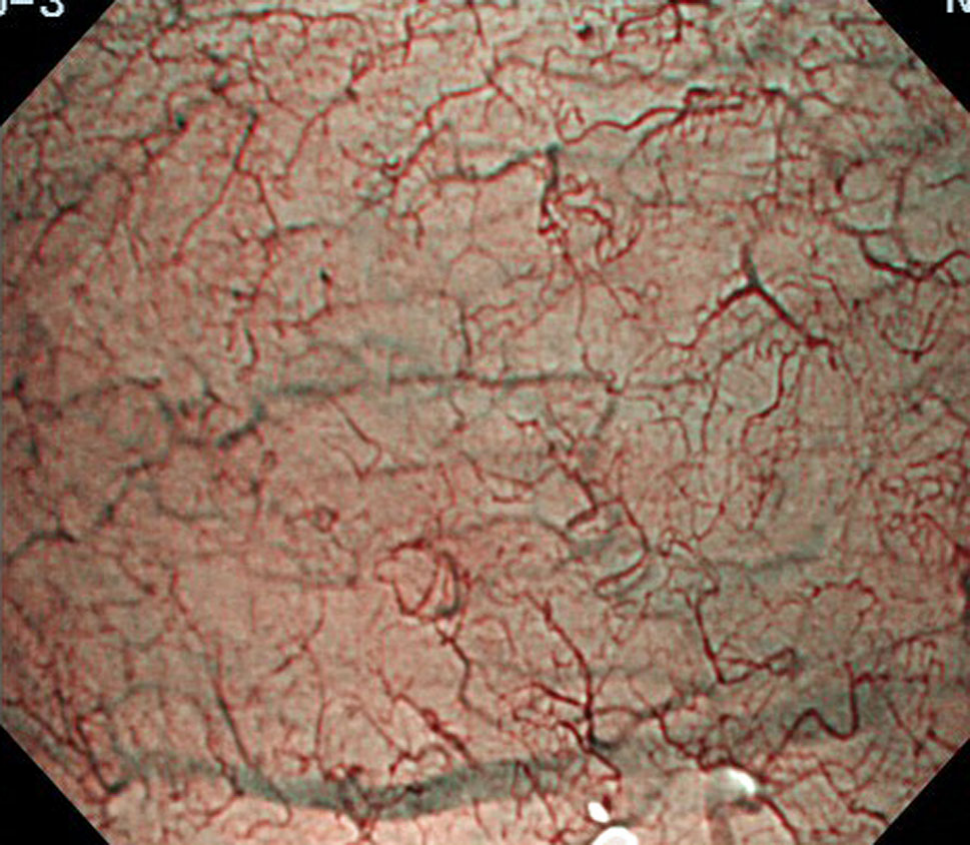
Flat-type mucosa: completely flat surface without a clear demarcation line and greenish thick vessels

These new classifications include the Japan Esophageal Society classification of BE [29], in which the mucosal and vascular patterns are described as either regular or irregular (Table 2), based on detailed definitions of regular and irregular in terms of mucosal and vascular shape or arrangement (Figs. 1, 2) (Table 3), thus making the findings easy to interpret. This classification also includes a flat pattern (Fig. 3) as a regular pattern corresponding to non-dysplastic histology [30]. A validation study conducted by 10 endoscopic image reviewers using 156 still images showed promising accuracy and inter-observer agreement (Table 1).

**Table 2:** Japan Esophageal Society classification of Barrett’s esophagus

Pattern	Visibility	Morphologic features	Regularity
Mucosal	Visible	Pit	Regular or irregular
		Non-pit	
	Invisible^a^		
Vascular	Visible	Net	Regular or irregular
		Non-net^b^	
	Invisible		

^a^Including a flat pattern

^b^Including normal-appearing long branching vessels and thick greenish vessels suggestive of a flat pattern

**Table 3:** Definition of regularity in Japan Esophageal Society classification of Barrett’s esophagus

Pattern	Regular	Irregular
Mucosal		
Form/size	Similar	Various
Arrangement	Regular	Irregular
Density	Low or same as surrounding area	High
White zone	Clearly visible and/or with homogeneous width	Obscure/invisible or heterogeneous width
Vascular		
Form	Similar or bending and branching gently or regularly	Various or bending and branching steeply or irregularly
Caliber change	Gradual	Abrupt
Location	Between or in mucosal patterns	Beyond of regardless of mucosal patterns
Flat pattern	Completely flat surface without a clear demarcation line. Greenish thick vessels and/or long branching vessels

## Diagnosis of cancer invasion depth

Correct preoperative staging is crucial, given that the patient’s treatment strategy is determined largely on the basis of cancer invasion depth. Non-magnified endoscopy is the primary modality for diagnosing gastrointestinal cancer, and is also helpful for diagnosing cancer invasion depth. Correlations between endoscopic macroscopic type and invasion depth of superficial EAC have been reported [31, 32], and previous studies showed that non-magnified endoscopy could accurately diagnose invasion depth in gastrointestinal cancers [33–36]. One study found that the overall correct diagnostic assessment of early esophageal cancers was high using either non-magnified endoscopy or endoscopic ultrasonography (EUS) with a 20-MHz mini-probe, with no significant differences between the two techniques (Table 4) [37]. Although its relative simplicity means that non-magnified endoscopy may be a good modality for diagnosing EAC invasion depth, the diagnosis is subjective, and more objective criteria are, therefore, needed.

**Table 4:** Diagnostic performances of non-magnified endoscopy and endoscopic ultrasonography for superficial esophageal adenocarcinoma (sensitivity and specificity for mucosal cancer)

Author	Country/year/sample size	Modality	Sensitivity	Specificity	Accuracy
May A [37]	Germany/2004/93	Non-magnified endoscopy	94	56	83
		EUS	91	48	79

*EUS* endoscopic ultrasonography

EUS can also be used to diagnose cancer invasion depth. Conventional EUS (7.5 MHz) can differentiate between advanced T3/T4 carcinomas and T1/T2 carcinomas in more than 80% of cases; however, accurate differentiation between mucosal and submucosal (SM) invasion is difficult [38–41]. However, EUS using a mini-probe (20 MHz) enables the esophageal wall to be imaged in nine layers, thus permitting the muscularis mucosa to be seen in greater detail. Mini-probe EUS can, therefore, be used to distinguish between mucosal and SM cancers, thereby improving staging accuracy.

A previous meta-analyses of EUS staging of superficial esophageal cancers showed favorable pooled values for mucosal cancer staging, with a sensitivity of 0.85 [95% confidence interval (CI) 0.82–0.88], specificity of 0.87 (95% CI 0.84–0.90), positive likelihood ratio of 6.62 (95%CI 3.6–12.12), and negative likelihood ratio of 0.20 (95%CI 0.14–0.30). The equivalent values for SM cancer staging were 0.86 for sensitivity (95%CI 0.82–0.89), 0.86 for specificity (95%CI 0.83–0.89), 5.13 for positive likelihood ratio (95%CI 3.36–7.82), and 0.17 for negative likelihood ratio (95%CI 0.09–0.30) [42].

However, when the results were limited to the diagnosis of EAC, the performance of EUS was not satisfactory (Table 5) [43–46] compared with its ability to diagnose esophageal squamous cell carcinoma and gastric cancer. Meta-analyses of the diagnostic accuracy of EUS for mucosal or SM micro-invasive esophageal squamous cell carcinoma showed a sensitivity of 0.87 (95%CI 0.81–0.92), specificity 0.94 (95%CI 0.88–0.98), positive likelihood ratio 11.6 (95%CI 5.4–24.7), and negative likelihood ratio 0.15 (95%CI 0.10–0.23) [47], with equivalent results for mucosal gastric cancer of sensitivity 0.87 (95%CI 0.81–0.92), specificity 0.75 (95%CI 0.62–0.84), positive likelihood ratio 3.4 (95%CI 2.3–5.0), and negative likelihood ratio 0.17 (95%CI 0.12–0.24) [48].

**Table 5:** Diagnostic performance of endoscopic ultrasonography for superficial esophageal adenocarcinoma (sensitivity and specificity for mucosal cancer)

Author	Country/year	Sample size	Sensitivity	Specificity	Accuracy
Thomas T [43]	UK/2010	46	94	67	85
Fernández-Sordo JO [44]	USA/2012	109	84	50	83
Bergeron EJ [45]	USA/2014	107	72	49	64
Dhupar R [46]	USA/2015	130	59	69	64

The poor diagnostic yield was probably caused by difficulties in diagnosing EAC in the distal part of the esophagus, given that the diagnostic accuracy for EAC in the distal part of the esophagus was significantly worse than that for EAC in the mid- and proximal parts of the esophagus (Table 6) [37, 49]. This emphasizes the fact that it is particularly difficult to achieve adequate water preparation in the distal esophagus by instilling fluid through the endoscopic channel, in addition to substantial motility that prevents dilatation of the distal esophagus from being maintained for longer periods.

**Table 6:** Diagnostic performance of endoscopic ultrasonography for superficial esophageal adenocarcinoma (sensitivity and specificity for mucosal cancer) with regard to imaging modality and lesion location

Author	Country/year/sample size	Modality	Location	Sensitivity	Specificity	Accuracy
May A [37]	Germany/2004/93	Non-magnified endoscopy	Distal	92	43	78
			Mid to proximal	97	91	95
		EUS	Distal	89	14	69
			Mid to proximal	94	91	93
Chemaly M [49]	France/2008/91	EUS	Distal	Not described	Not described	48
			Mid to proximal	Not described	Not described	87

*EUS* endoscopic ultrasonography

## Endoscopic resection

**Fig. 4:**
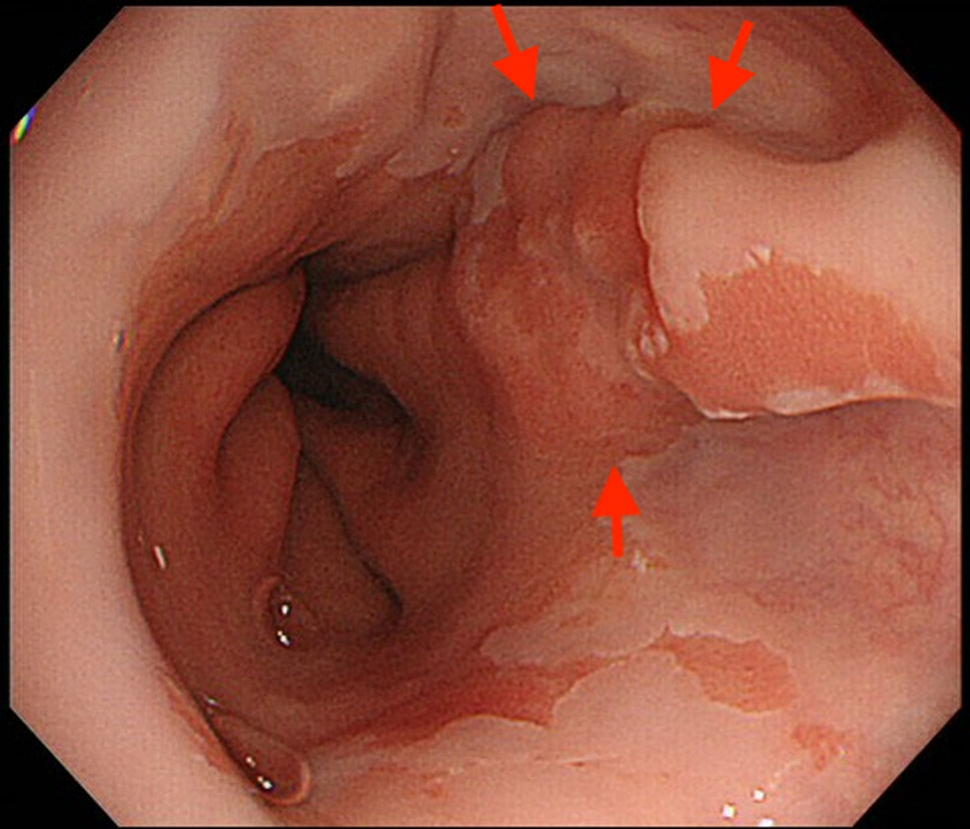
IIa type esophagogastric junctional cancer

**Fig. 5:**
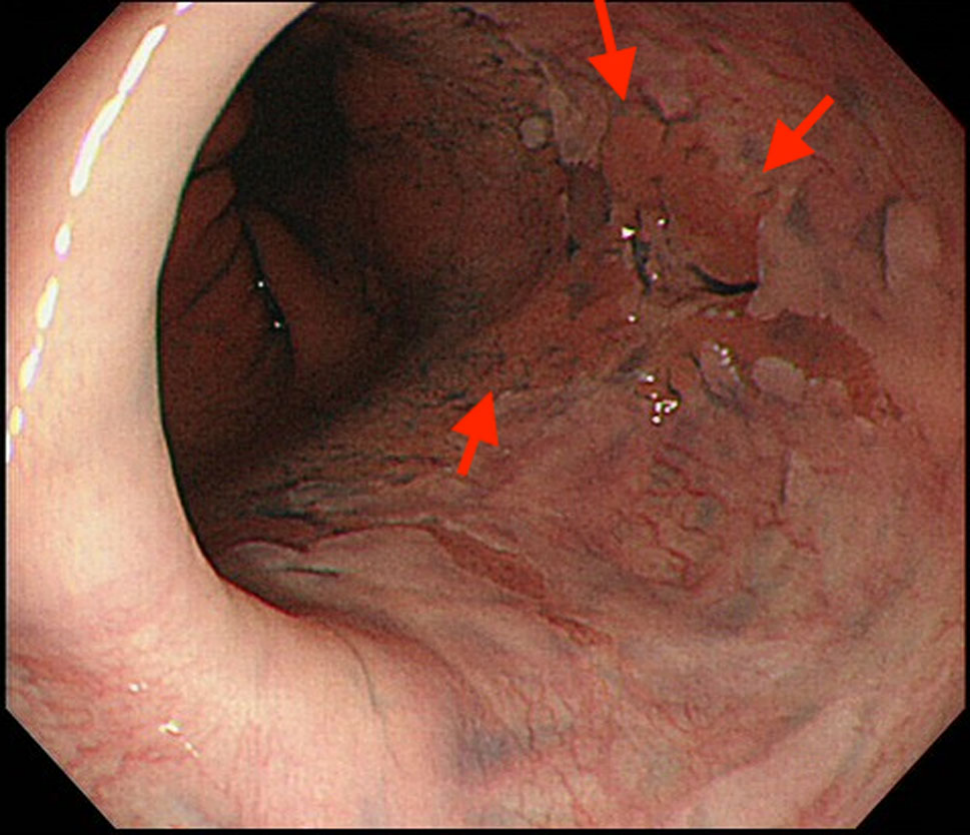
IIa type esophagogastric junctional cancer with indigo carmine staining

**Fig. 6:**
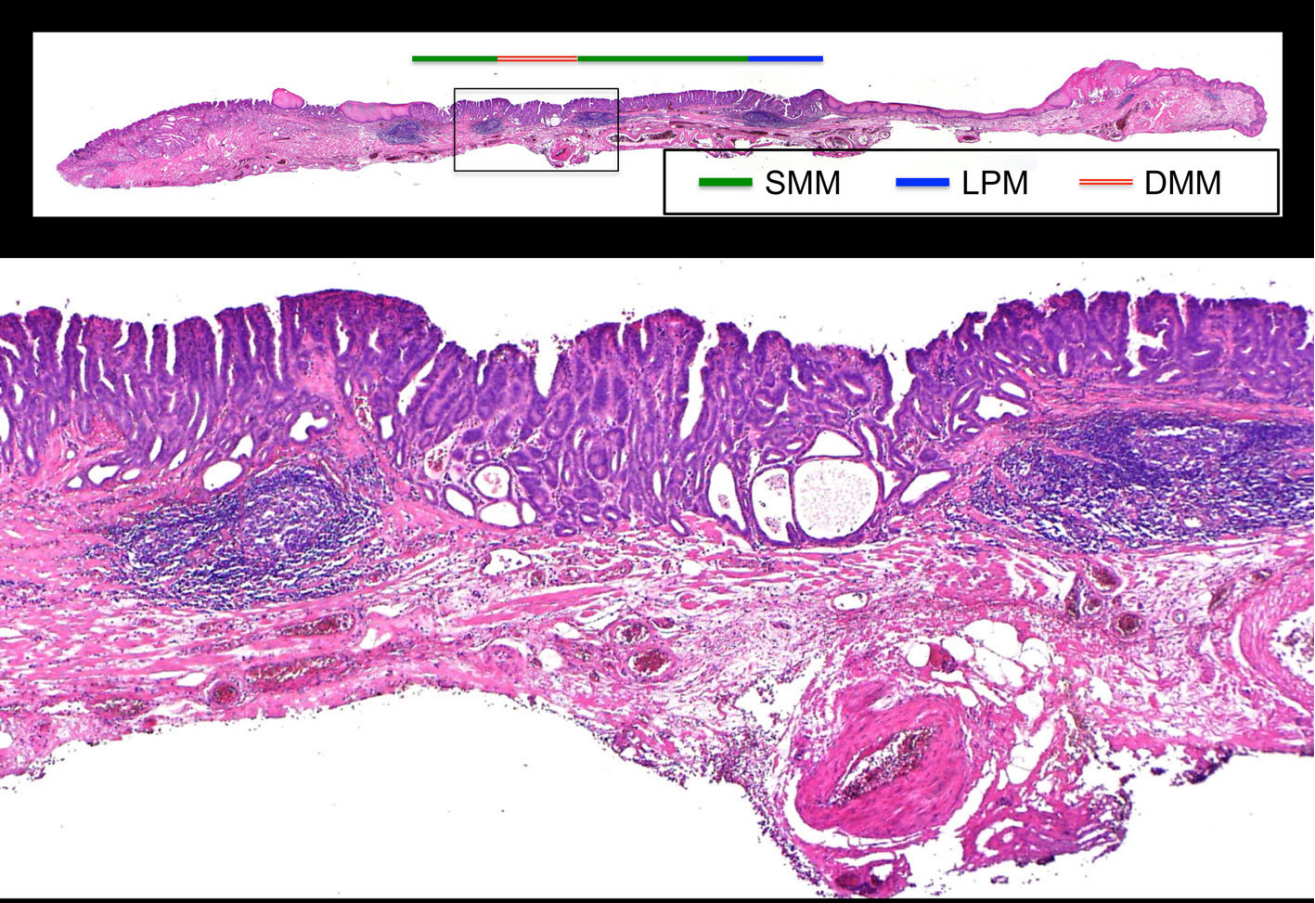
Histology of resected specimen showed deep muscularis mucosa invasion of cancer. *SMM* superficial muscularis mucosa, *LPM* lamina propria, *DMM* deep muscularis mucosa

**Fig. 7:**
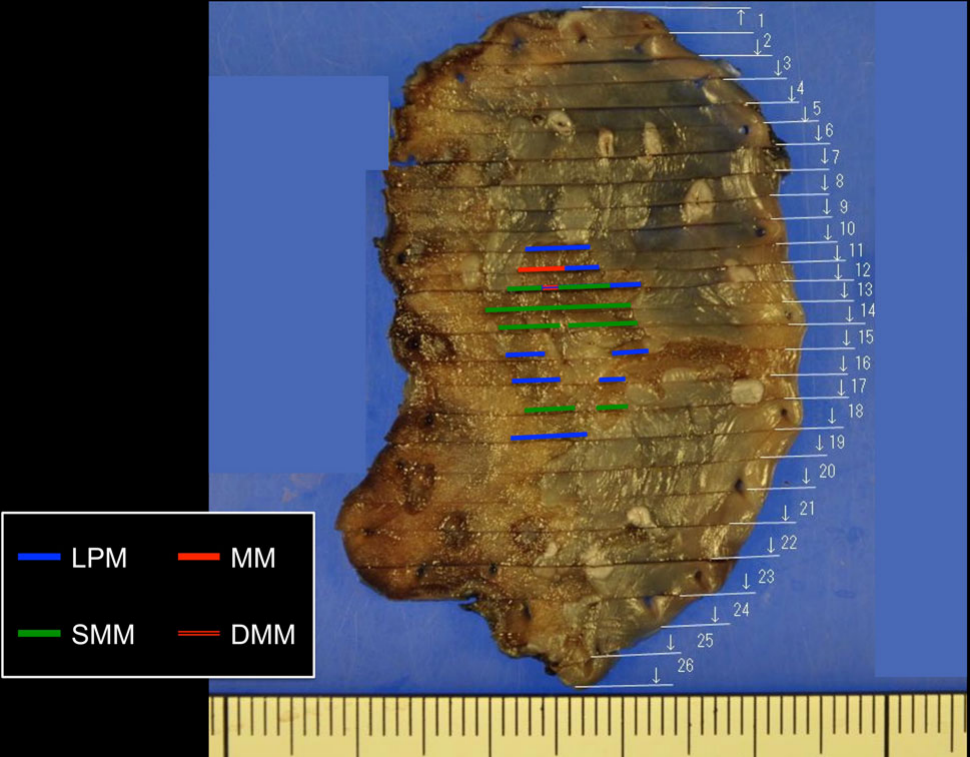
Mapping of the cancer. *SMM* superficial muscularis mucosa, *LPM* lamina propria, *DMM* deep muscularis mucosa, *MM* muscularis mucosa

Endoscopic resection has recently been suggested as a staging modality for superficial gastrointestinal cancers, based on the limited accuracies of EUS and non-magnified endoscopy. Endoscopic resection, in the form of endoscopic mucosal resection (EMR) and endoscopic submucosal dissection (ESD), allows for removal of visible lesions and histologic assessment of the resected tissue, thus facilitating accurate diagnostic staging of the disease (Figs. 4, 5, 6, 7) [50, 51].

The various modalities of EMR include the use of a transparent cap, two-channel endoscope, and ligation. However, these modalities are limited with respect to resection size, and large lesions must be resected in several fragments. Histological assessment of cancer invasion depth can be inaccurate if lesions are resected in small fragments, and histologic evaluation of several specimens does not allow the outer margins of the neoplastic area to be identified, and complete resection, therefore, cannot be confirmed. In addition, piecemeal resection of early neoplasia in BE is associated with a high local recurrence rate, probably because of small remnants of neoplastic tissue left in situ [52–55]. ESD provides larger specimens than EMR, thus allowing more precise histological analysis and higher en bloc and curative resection rates, and potentially reducing the incidence of recurrence. A recent meta-analysis of non-randomized studies showed that ESD of early gastrointestinal tumors was superior to EMR in terms of en bloc and curative resection rates, but was more time consuming and associated with higher rates of bleeding and perforation [56].

Several studies have reported on the use of ESD for EAC and esophagogastric junction cancer [57–66]. In general, ESD is associated with favorable outcomes with acceptable en bloc resection and complication rates. However, the curative resection rate, defined as en bloc resection with cancer-free margins and minimal risk of metastasis, limited to EAC at the esophagogastric junction, was significantly lower than those for cardia and non-cardia gastric cancers (Table 7) [57, 66]. One cause of incomplete resection of esophagogastric junction EAC was positive lateral margins caused by sub-epithelial progression of the tumor proximally, which were hard to recognize before treatment, while the low accuracy of diagnosing cancer invasion depth before treatment and high lymphovascular involvement, confirmed in resected specimens, were also contributory factors.

**Table 7:** Outcomes of endoscopic submucosal dissection for esophagogastric junctional cancer with regard to location

Author	Location	Complete resection^a^	Curative resection^b^
Osumi H [66]	Esophagus	100% (55/55)	62% (34/55)
	Cardia	100% (87/87)	82% (71/87)
Hoteya S [57]	Esophagus	64% (16/25)	48% (12/25)
	Cardia	96% (99/103)	81% (83/103)

^a^Complete resection: en bloc resection with cancer-free margins

^b^Curative resection: complete resection with low risk of metastasis

## Risk of metastasis

The risk of metastasis after endoscopic resection for gastrointestinal cancers is evaluated based on histologic findings of the resected specimen. Studies of esophagectomy specimens have indicated a low risk of 0.0–1.3% for mucosal EAC [67–69], thus providing the rationale for endoscopic treatment of mucosal EAC with curative intent.

The frequency of metastasis in EAC is known to increase with increasing depth of tumor invasion into the SM [70–72]. SM1 cancer, i.e., cancer invading the shallow part of the SM, remains the most controversial, with some studies reporting a relevant incidence of lymph node metastasis even in SM1 cancers [73–75]. However, when the rate of metastasis is stratified by pathologic findings, SM1 cancers without risk factors such as lymphovascular involvement and a poorly differentiated component have very low rates [76–78]. Some studies [79, 80] have accordingly suggested that a subgroup of SM cancers could be adequately treated by endoscopic resection.

European guidelines [80] indicate that endoscopic resection appears to be curative for well- or moderately differentiated mucosal cancers without lymphatic or vascular invasion, and that these criteria might be extended to lesions with invasion into the SM (≤ 500 μm), namely to low-risk tumors (well or moderately differentiated, without lymphovascular involvement, < 3 cm) (Table 8). A recent study in Japan [81] validated these criteria, showing no metastases (0/186 lesions) in patients with mucosal cancer without lymphovascular involvement and a poorly differentiated component, or in patients with SM cancer (≤ 500 μm) without lymphovascular involvement, a poorly differentiated component, and ≤ 30 mm (0/32 lesions).

**Table 8:** Assessment of metastasis risk based on histology of endoscopically resected specimen

European guideline [80]	Curative for	Curative criteria might be extended to
	Mucosal cancer Well or moderately differentiated Lymphovascular involvement(−)	Submucosal cancer (≤ 500 μm) Well or moderately differentiated Lymphovascular involvement(−) Tumor size < 3 cm
Report from Japan Ishihara R [81]	Very low risk (no metastasis in 186 cancers)	Low risk (no metastasis in 32 cancers)
	Mucosal cancer Poorly differentiated component (−) Lymphovascular involvement (−)	Submucosal cancer (≤ 500 μm) Poorly differentiated component (−) Lymphovascular involvement (−) Tumor size ≤ 3 cm

## Future perspectives

Recent advances in endoscopic technologies have provided various tools for the management of gastrointestinal cancers. Previous studies showed the utility of such tools for the early detection and accurate staging of cancers. However, most of these studies were retrospective and limited by small sample sizes. Prospective, multicenter studies are, therefore, needed to provide more reliable evidence and facilitate the use of these tools in clinical practice.

## References

[1] DeMeester SR (2006). Adenocarcinoma of the esophagus and cardia: a review of the disease and its treatment. Ann Surg Oncol.

[2] Shaheen NJ, Richter JE (2009). Barrett’s oesophagus. Lancet.

[3] Brown LM, Devesa SS, Chow WH (2008). Incidence of adenocarcinoma of the esophagus among white Americans by sex, stage, and age. J Natl Cancer Inst.

[4] Wu JC (2008). Gastroesophageal reflux disease: an Asian perspective. J Gastroenterol Hepatol.

[5] Hongo M, Nagasaki Y, Shoji T (2009). Epidemiology of esophageal cancer: orient to occident. Effects of chronology, geography and ethnicity. J Gastroenterol Hepatol.

[6] Gillison EW, Powell J, Mcconkey CC (2002). Surgical workload and outcome after resection for carcinoma of the oesophagus and cardia. Br J Surg.

[7] Inadomi JM, Sampliner R, Lagergren J (2003). Screening and surveillance for Barrett esophagus in high-risk groups: a cost-utility analysis. Ann Intern Med.

[8] Sharma P, Sidorenko EI (2005). Are screening and surveillance for Barrett’s oesophagus really worthwhile?. Gut.

[9] van Sandick JW, Bartelsman JF, van Lanschot JJ (2000). Surveillance of Barrett’s oesophagus: physicians’ practices and review of current guidelines. Eur J Gastroenterol Hepatol.

[10] Falk GW, Ours TM, Richter JE (2000). Practice patterns for surveillance of Barrett’s esophagus in the United States. Gastrointest Endosc.

[11] Gross CP, Canto MI, Hixson J (1999). Management of Barrett’s esophagus: a national study of practice patterns and their cost implications. Am J Gastroenterol.

[12] WangKK SamplinerRE (2008). Updated guidelines 2008 for the diagnosis, surveillance and therapy of Barrett’s esophagus. Am J Gastroenterol.

[13] Abrams JA, Kapel RC, Lindberg GM (2009). Adherence to biopsy guidelines for Barrett’s esophagus surveillance in the community setting in the United States. Clin Gastroenterol Hepatol..

[14] Gono K, Obi T, Yamaguchi M (2004). Appearance of enhanced tissue features in narrow-band endoscopic imaging. J Biomed Opt.

[15] Sharma P, Hawes RH, Bansal A (2013). Standard endoscopy with random biopsies versus narrow band imaging targeted biopsies in Barrett’s oesophagus: a prospective, international, randomised controlled trial. Gut.

[16] Sharma P, Bansal A, Mathur S (2006). The utility of a novel narrow band imaging endoscopy system in patients with Barrett’s esophagus. Gastrointest Endosc.

[17] Kara MA, Ennahachi M, Fockens P (2006). Detection and classification of the mucosal and vascular patterns (mucosal morphology) in Barrett’s esophagus by using narrow band imaging. Gastrointest Endosc.

[18] Singh R, Anagnostopoulos GK, Yao K (2008). Narrow-band imaging with magnification in Barrett’s esophagus: validation of a simplified grading system of mucosal morphology patterns against histology. Endoscopy.

[19] Hamamoto Y, Endo T, Nosho K (2004). Usefulness of narrow-band imaging endoscopy for diagnosis of Barrett’s esophagus. J Gastroenterol.

[20] Goda K, Tajiri H, Ikegami M (2007). Usefulness of magnifying endoscopy with narrow band imaging for the detection of specialized intestinal metaplasia in columnar-lined esophagus and Barrett’s adenocarcinoma. Gastrointest Endosc.

[21] Anagnostopoulos GK, Yao K, Kaye P (2007). Novel endoscopic observation in Barrett’s oesophagus using high resolution magnification endoscopy and narrow band imaging. Aliment Pharmacol Ther.

[22] Alvarez Herrero L, Curvers WL, Bansal A (2009). Zooming in on Barrett oesophagus using narrow-band imaging: an international observer agreement study. Eur J Gastroenterol Hepatol.

[23] Singh M, Bansal A, Curvers WL (2011). Observer agreement in the assessment of narrowband imaging system surface patterns in Barrett’s esophagus: a multicenter study. Endoscopy.

[24] Silva FB, Dinis-Ribeiro M, Vieth M (2011). Endoscopic assessment and grading of Barrett’s esophagus using magnification endoscopy and narrow-band imaging: accuracy and interobserver agreement of different classification systems (with videos). Gastrointest Endosc.

[25] Curvers WL, Bohmer CJ, Mallant-Hent RC (2008). Mucosal morphology in Barrett’s esophagus: interobserver agreement and role of narrow band imaging. Endoscopy.

[26] Curvers W, Baak L, Kiesslich R (2008). Chromoendoscopy and narrow-band imaging compared with high-resolution magnification endoscopy in Barrett’s esophagus. Gastroenterology.

[27] Baldaque-Silva F, Marques M, Lunet N (2013). Endoscopicassessment and grading of Barrett’s esophagus using magnification endoscopy and narrow band imaging: impact of structured learning and experience on the accuracy of the Amsterdam classification system. Scand J Gastroenterol.

[28] Sharma P, Bergman J, Goda K (2016). Development and validation of a classification system to identify high-grade dysplasia and esophageal adenocarcinoma in Barrett’s esophagus using narrow-band imaging. Gastroenterology.

[29] Goda K, Fujisaki J, Ishihara R, et al. Newly developed magnifying endoscopic classification of the Japan Esophageal Society to identify superficial Barrett’s esophagus-related neoplasms. Esophagus. **[Epub ahead of print]**. 10.1007/s10388-018-0623-y PMC6021472 29923024

[30] Kato M, Goda K, Shimizu Y (2017). Image assessment of Barrett’s esophagus using the simplified narrow band imaging classification. J Gastroenterol.

[31] Pech O, Gossner L, Manner H (2007). Prospective evaluation of the macroscopic types and location of early Barrett’s neoplasia in 380 lesions. Endoscopy.

[32] Oda I, Abe S, Kusano C (2011). Correlation between endoscopic macroscopic type and invasion depth for early esophagogastric junction adenocarcinomas. Gastric Cancer.

[33] Abe S, Oda I, Shimazu T (2011). Depth-predicting score for differentiated early gastric cancer. Gastric Cancer.

[34] Tsujii Y, Kato M, Inoue T (2015). Integrated diagnostic strategy for the invasion depth of early gastric cancer by conventional endoscopy and EUS. Gastrointest Endosc.

[35] Choi J, Kim SG, Im JP (2010). Comparison of endoscopic ultrasonography and conventional endoscopy for prediction of depth of tumor invasion in early gastric cancer. Endoscopy.

[36] Nagahama T, Yao K, Imamura K (2017). Diagnostic performance of conventional endoscopy in the identification of submucosal invasion by early gastric cancer: the “non-extension sign” as a simple diagnostic marker. Gastric Cancer.

[37] May A, Günter E, Roth F (2004). Accuracy of staging in early oesophageal cancer using high resolution endoscopy and high resolution endosonography: a comparative, prospective, and blinded trial. Gut.

[38] Greenberg J, Durkin M, van Drunen M (1994). Computed tomography or endoscopic ultrasonography in preoperative staging of gastric and esophageal tumors. Surgery.

[39] Meining A, Dittler HJ, Wolf A (2002). You get what you expect? A critical appraisal of imaging methodology in endosonographic cancer staging. Gut.

[40] Hiele M, De Leyn P, Schurmans P (1997). Relation between endoscopic ultrasound findings and outcome of patients with tumors of the esophagus or esophagogastric junction. Gastrointest Endosc.

[41] Kelly S, Harris KM, Berry E (2001). A systematic review of the staging performance of endoscopic ultrasound in gastro-oesophageal carcinoma. Gut.

[42] Thosani N, Singh H, Kapadia A (2012). Diagnostic accuracy of EUS in differentiating mucosal versus submucosal invasion of superficial esophageal cancers: a systematic review and meta-analysis. Gastrointest Endosc.

[43] Thomas T, Gilbert D, Kaye PV (2010). High-resolution endoscopy and endoscopic ultrasound for evaluation of early neoplasia in Barrett’s esophagus. Surg Endosc.

[44] Fernández-Sordo JO, Konda VJ, Chennat J (2012). Is Endoscopic Ultrasound (EUS) necessary in the pre-therapeutic assessment of Barrett’s esophagus with early neoplasia?. J Gastrointest Oncol.

[45] Bergeron EJ, Lin J, Chang AC (2014). Endoscopic ultrasound is inadequate to determine which T1/T2 esophageal tumors are candidates for endoluminal therapies. J Thorac Cardiovasc Surg.

[46] Dhupar R, Rice RD, Correa AM (2015). Endoscopic ultrasound estimates for tumor depth at the gastroesophageal junction are inaccurate: implications for the liberal use of endoscopic resection. Ann Thorac Surg.

[47] Ishihara R, Matsuura N, Hanaoka N (2017). Endoscopic imaging modalities for diagnosing invasion depth of superficial esophageal squamous cell carcinoma: a systematic review and meta-analysis. BMC Gastroenterol.

[48] Mocellin S, Pasquali S (2015). Diagnostic accuracy of endoscopic ultrasonography (EUS) for the preoperative locoregional staging of primary gastric cancer. Cochrane Database Syst Rev.

[49] Chemaly M, Scalone O, Durivage G (2008). Miniprobe EUS in the pretherapeutic assessment of early esophageal neoplasia. Endoscopy.

[50] Bennett C, Vakil N, Bergman J (2012). Consensus statements for management of Barrett’s dysplasia and early-stage esophageal adenocarcinoma, based on a Delphi process. Gastroenterology.

[51] Fitzgerald RC, di Petro M, Ragunath K (2014). British society of gastroenterology guidelines on the diagnosis and management of Barrett’s oesophagus. Gut.

[52] Ell C, May A, Gossner L (2000). Endoscopic mucosal resection of early cancer and high-grade dysplasia in Barrett’s esophagus. Gastroenterology.

[53] Peters FP, Kara MA, Rosmolen WD (2005). Endoscopic treatment of high-grade dysplasia and early stage cancer in Barrett’s esophagus. Gastrointest Endosc.

[54] Ell C, May A, Pech O (2007). Curative endoscopic resection of early esophageal adenocarcinomas (Barrett’s cancer). Gastrointest Endosc.

[55] Pech O, Behrens A, May A (2008). Long-term results and risk factor analysis for recurrence after curative endoscopic therapy in 349 patients with high-grade intraepithelial neoplasia and mucosal adenocarcinoma in Barrett’s oesophagus. Gut.

[56] Cao Y, Liao C, Tan A (2009). Meta-analysis of endoscopic submucosal dissection versus endoscopic mucosal resection for tumors of the gastrointestinal tract. Endoscopy.

[57] Hoteya S, Matsui A, Iizuka T (2013). Comparison of the clinicopathological characteristics and results of endoscopic submucosal dissection for esophagogastric junction and non-junctional cancers. Digestion.

[58] Ikeda K, Isomoto H, Oda H (2009). Endoscopic submucosal dissection of a minute intramucosal adenocarcinoma in Barrett’s esophagus. Dig Endosc.

[59] Probst A, Aust D, Märkl B (2015). Early esophageal cancer in Europe: endoscopic treatment by endoscopic submucosal dissection. Endoscopy.

[60] Chevaux JB, Piessevaux H, Jouret-Mourin A (2015). Clinical outcome in patients treated with endoscopic submucosal dissection for superficial Barrett’s neoplasia. Endoscopy.

[61] Terheggen G, Horn EM, Vieth M (2017). A randomised trial of endoscopic submucosal dissection versus endoscopic mucosal resection for early Barrett’s neoplasia. Gut.

[62] Yoshinaga S, Gotoda T, Kusano C (2008). Clinical impact of endoscopic submucosal dissection for superficial adenocarcinoma located at the esophagogastric junction. Gastrointest Endosc.

[63] Hirasawa K, Kokawa A, Oka H (2010). Superficial adenocarcinoma of the esophagogastric junction: long-term results of endoscopic submucosal dissection. Gastrointest Endosc.

[64] Kakushima N, Yahagi N, Fujishiro M (2006). Efficacy and safety of endoscopic submucosal dissection for tumors of the esophagogastric junction. Endoscopy.

[65] Omae M, Fujisaki J, Horiuchi Y (2013). Safety, efficacy, and long-term outcomes for endoscopic submucosal dissection of early esophagogastric junction cancer. Gastric Cancer.

[66] Osumi H, Fujisaki J, Omae M (2017). Clinicopathological features of Siewert type II adenocarcinoma: comparison of gastric cardia adenocarcinoma and Barrett’s esophageal adenocarcinoma following endoscopic submucosal dissection. Gastric Cancer.

[67] Stein HJ, Feith M, Bruecher BL (2005). Early esophageal cancer: pattern of lymphatic spread and prognostic factors for long-term survival after surgical resection. Ann Surg.

[68] Leers JM, Demeester SR, Oezcelik A (2011). The prevalence of lymph node metastases in patients with T1 esophageal adenocarcinoma a retrospective review of esophagectomy specimens. Ann Surg.

[69] Barbour AP, Jones M, Brown I (2010). Risk stratification for early esophageal adenocarcinoma: analysis of lymphatic spread and prognostic factors. Ann Surg Oncol.

[70] Westerterp M, Koppert LB, Buskens CJ (2005). Outcome of surgical treatment for early adenocarcinoma of the esophagus or gastro-esophageal junction. Virchows Arch.

[71] Buskens CJ, Westerterp M, Lagarde SM (2004). Prediction of appropriateness of local endoscopic treatment for high-grade dysplasia and early adenocarcinoma by EUS and histopathologic features. Gastrointest Endosc.

[72] Sepesi B, Watson TJ, Zhou D (2010). Are endoscopic therapies appropriate for superficial submucosal esophageal adenocarcinoma? An analysis of esophagectomy specimens. J Am Coll Surg.

[73] Griffin SM, Burt AD, Jennings NA (2011). Lymph node metastasis in early esophageal adenocarcinoma. Ann Surg.

[74] Bollschweiler E, Baldus SE, Schroder W (2006). High rate of lymph-node metastasis in submucosal esophageal squamous cell carcinomas and adenocarcinomas. Endoscopy.

[75] Manner H, Pech O, Heldmann Y (2013). Efficacy, safety, and long-term results of endoscopic treatment for early stage adenocarcinoma of the esophagus with low-risk sm1 invasion. Clin Gastroenterol Hepatol.

[76] Alvarez HL, Pouw RE, van Vilsteren FG (2010). Risk of lymph node metastasis associated with deeper invasion by early adenocarcinoma of the esophagus and cardia: study based on endoscopic resection specimens. Endoscopy.

[77] Manner H, Pech O, Heldmann Y (2015). The frequency of lymph node metastasis in early-stage adenocarcinoma of the esophagus with incipient submucosal invasion (pT1b sm1) depending on histological risk patterns. Surg Endosc.

[78] Greene CL, Worrell SG, Attwood SE (2016). Emerging concepts for the endoscopic management of superficial esophageal adenocarcinoma. J Gastrointest Surg.

[79] Mohiuddin K, Dorer R, El Lakis MA (2016). Outcomes of surgical resection of T1bN0 esophageal cancer and assessment of endoscopic mucosal resection for identifying low-risk cancers appropriate for endoscopic therapy. Ann Surg Oncol.

[80] Pimentel-Nunes P, Dinis-Ribeiro M, Ponchon T (2015). Endoscopic submucosal dissection: European society of gastrointestinal endoscopy (ESGE) guideline. Endoscopy.

[81] Ishihara R, Oyama T, Abe S (2017). Risk of metastasis in adenocarcinoma of the esophagus: a multicenter retrospective study in a Japanese population. J Gastroenterol.

